# Efficacy of Wound Instillation With Bupivacaine Through a Wound Catheter for Postoperative Analgesia in Laparotomy Wounds in Comparison With Conventional Intravenous Analgesics

**DOI:** 10.7759/cureus.38914

**Published:** 2023-05-11

**Authors:** Pankaja S S, Deepanjali Shetty, C. p Madhu, Thulasi Vasudevaiah, Akash M V

**Affiliations:** 1 Department of General Surgery, Jagadguru Sri Shivarathreeshwara (JSS) Medical College and Hospital, JSS Academy of Higher Education and Research, Mysuru, IND; 2 Department of General Surgery, Adichunchanagiri Institute of Medical Sciences, Adichunchanagiri University, BG Nagara, IND

**Keywords:** wound catheter instillation, postoperative analgesia, intravenous analgesics, bupivacaine, midline laparotomy, postoperative pain

## Abstract

Introduction

A laparotomy can cause severe postoperative pain, which, if treated adequately, can result in reduced incidence of lung atelectasis and ileus promoting early mobilization and faster recovery and in turn reducing the duration of hospital stays. Hence, effective postoperative analgesia is important to reduce postoperative stress and improve early surgical outcomes. Therefore, the hypothesis is based on the fact that following a midline laparotomy, instillation of local anaesthetic agent 0.25% bupivacaine through a wound catheter placed in the subcutaneous plane may provide better analgesia compared to the conventional intravenous analgesics and improve the early surgical outcomes.

Methodology

A prospective, comparative, quasi-experimental study was conducted on 80 patients planned for emergency or elective midline laparotomy procedures over a period of 18 months, who were randomly distributed into two groups of 40 each. The bupivacaine group consisted of 40 patients who received 10ml of 0.25% bupivacaine instilled through a wound catheter placed in the subcutaneous plane following a midline laparotomy. This was repeated every six hours for the first 24 hours followed by every 12 hours for the next 24 hours. The conventional intravenous (IV) analgesics group involved 40 patients who received conventional IV analgesics routinely used. Pain scores were recorded every four hours for 60 hours using the visual analogue scale (VAS) and dynamic visual analogue scale (DVAS). The parameters assessed were mean VAS and DVAS scores, number of rescue analgesic demands, cumulative rescue analgesic requirement, and early surgical outcomes. Wound complications were also assessed.

Results

Both groups shared similar demographic characteristics in terms of age, gender, comorbidities, and duration of operation. In comparison to patients who got standard IV analgesics, those who received 0.25% bupivacaine had improved postoperative analgesia. Between the two groups, there were statistically significant results in the number of rescue analgesic demands in the first 24 hours, but in the next 24 hours, it was statistically insignificant. The study also showed that bupivacaine instillation led to a significant decrease in postoperative lung complications and the length of hospital stays; however as hypothesised, it did not improve early surgical outcomes.

Conclusion

This modality, the instillation of bupivacaine through a wound catheter, is an efficient and technically simple method to provide optimal postoperative analgesia. It substantially reduces the need for systemic analgesics and can potentially avoid their related side effects. Hence, the armamentarium of multimodal analgesia can therefore include this method of delivering postoperative analgesia.

## Introduction

The International Association for the Study of Pain and the World Health Organization both regard access to pain relief as a fundamental human right [1]. Postoperatively, patients experience acute pain in around 75% of cases [2]. Only about half of these patients report adequate postoperative pain relief [3]. Systemic analgesics opioids and non-steroidal anti-inflammatory drugs (NSAIDs) have always been the cornerstone of care for immediate postoperative pain. However, there has been a recent increase in morbidity and mortality linked to opioid/NSAID abuse. This has put focus on the requirement for additional research into creating novel pain management protocols to help employ a multimodal strategy [4].

Major operations like laparotomies cause significant postoperative pain, which, if treated adequately, can result in less incidence of lung atelectasis and ileus, promoting early mobilisation and faster recovery and in turn reducing the duration of hospital stays. The cardiovascular (thromboembolic events, myocardial infarction), renal (oliguria, urinary retention), and pulmonary (infection, hypoventilation, diminished vital capacity) systems are just a few of the organ systems that can be negatively impacted by ineffective treatment of acute pain. Early postoperative discomfort seems to be linked to prolonged analgesic use both during and after hospitalisation, which may trigger the onset of chronic pain [5]. Between 2% and 10% of adults experience this severe, chronic postoperative pain [6]. Hence, appropriate postoperative analgesia is essential from the perspectives of both patients and medical professionals, as it can enhance surgical results [7].

After a specific surgery, an ensured technique in which the surgeon promptly inserts a catheter in the surgical site to infuse a local anaesthetic agent may help improve postoperative analgesia. It is technically efficacious and significantly lessens the need for NSAIDs and opioids and the negative effects that go along with it. There have been inconsistent reports regarding the overall efficacy and the risk-benefit of this modality, despite the fact that numerous publications and small trials have examined the use of continuous instillation through wound catheters in a variety of surgical procedures [8].

The hypothesis is based on the fact that following a midline laparotomy, the instillation of local anaesthetic 0.25% bupivacaine through a wound catheter placed in the subcutaneous plane may provide better analgesia compared to the conventional intravenous (IV) analgesics routinely used. Consequently, the goal of this study is to evaluate the effectiveness of local anaesthetic bupivacaine (0.25%) in wound instillation through a wound catheter for postoperative analgesia in laparotomy wounds, to compare the efficacy of instillation of local anaesthetic bupivacaine (0.25%) through a wound catheter with conventional IV analgesics for postoperative analgesia in laparotomy wounds, and to assess the advantages of alleviating early postoperative pain and study its effect on surgical outcomes.

## Materials and methods

A prospective, comparative, quasi-experimental study was carried out on 80 patients scheduled for emergency or elective midline laparotomy procedures in the Department of General Surgery at JSS Hospital, Mysuru over the course of 18 months after receiving approval from the Institutional Ethics Committee (IEC) with IEC number JSS/MC/PG/5156/2020-21. The study population was divided into two groups of 40 each by simple random sampling. Patients who were excluded were allergic to local anaesthetic bupivacaine, had a high risk of having faecal peritonitis, those planned for epidural anaesthesia, and those whose pain level could not be assessed (patients with a history of psychiatric illness or postoperative endotracheal tube in situ). Written informed consent was received from the participants, and only the investigator gathered the perioperative data. Prior to surgery, all patients received instruction on how to rate the static visual analogue scale (VAS) illustrating pain at rest and dynamic visual analogue scale (DVAS) illustrating pain at coughing consisting of a 10-cm scale with 0 denoting no pain and 10 denoting the most excruciating pain imaginable.

Group 1 was considered a bupivacaine group in which at the end of the surgical procedure a wound catheter (infant feeding tube no 8) was placed in the subcutaneous plane by the surgeon before closing the surgical incision. The wound catheter used was an infant feeding tube, 8 Fr, colour code-blue, with an outer diameter of 2.7mm and length of 51 cm and made up of medical grade polyvinyl chloride. Its distal end had two pre-existing lateral holes. Additional holes were made with sterile scissors, which were equidistant from each other, along their length to cover the laparotomy wound. At the end of the laparotomy wound, this infant feeding tube was fixed using the Mersilk 2-0 suture material (Ethicon, Edinburgh, Scotland). An infant feeding tube was selected for economic reasons, and it is easily available as other wound catheters or epidural catheters cost marginally more compared to infant feeding tubes for the same function. These patients received 10 ml of 0.25% bupivacaine through the catheter after the closure of the skin incision, then every six hours for the first 24 hours followed by every 12 hours for the next 24 hours. The wound catheter was removed after 48 hours. Group 2 was considered a conventional IV analgesics group consisting of 40 patients who received only conventional IV analgesics i.e., injection of tramadol 50mg or diclofenac sodium 75mg (in 100 ml of normal saline as IV slow infusion, three times a day).

Pain score (VAS/DVAS) at "0" hour was noted after extubation (when the patient became alert) and then subsequently every four hours for 60 hours. Rescue analgesia with an injection of tramadol (50 mg in 100 ml NS, as slow IV infusion) was given if the VAS/DVAS surpassed 5 at any point in time.

The study was assessed by the following parameters: Mean VAS and DVAS every four hours over 60 hours, number of demands for rescue analgesia, cumulative rescue analgesic requirement, time taken to start orally, to first pass flatus, for first bowel movement, and for independent ambulation, the occurrence of postoperative lung complications, surgical site complications, and length of hospital stays. Statistical analysis was performed using IBM SPSS Statistics for Windows, Version 26 (Released 2019; IBM Corp., Armonk, New York, United States).

## Results

**Figure 1:**
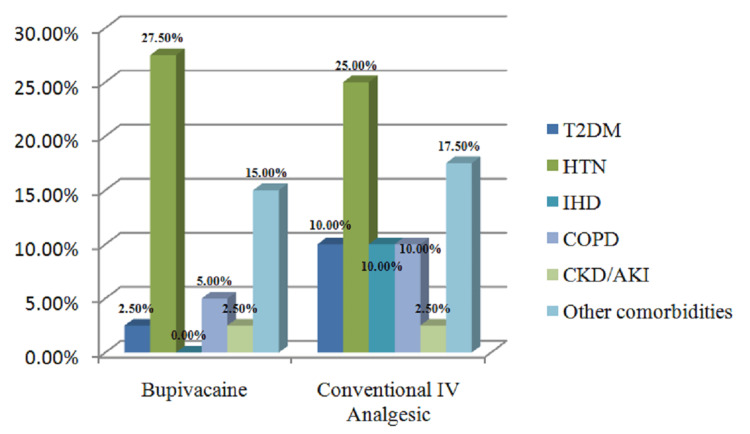
Distribution of comorbidities in each group. T2DM: Type 2 diabetes mellitus, HTN: hypertension, IHD: ischaemic heart disease, COPD: chronic obstructive pulmonary disease, CKD: chronic kidney disease, AKI: acute kidney injury, IV: intravenous

Regarding demographic information and duration of surgery, the two groups, bupivacaine and traditional IV analgesics, were equivalent. The mean age of the bupivacaine group and the conventional IV analgesics group was 48.02±15.7 years and 52.35±18.1 years, respectively. Demographically, the study population was predominantly male in both groups showing 77.5% in the bupivacaine group and 80% in the conventional IV analgesics group. The most common surgical procedure performed was laparotomy with Graham’s omentoplasty, with 23 (57.5%) in the bupivacaine group, whereas 14 (35%) in the conventional IV analgesics group. The length of the laparotomy wound was approximately 15-20 cm and there was no fixed length, as the length depended on the primary pathology. The average duration of surgery was 188.1 ± 55.2 (mins) and 175.3 ± 51.0 (mins) in the bupivacaine and conventional IV analgesics groups, respectively. The distribution of comorbidities in each group is demonstrated in Figure 1.

The postoperative pain assessment was recorded using the VAS and DVAS which is demonstrated in Table 1 and Table 2. In the present study, based on the VAS, the mean pain score over 60 hours was 2.39 ± 0.21 in the bupivacaine group, and in the conventional IV analgesics group, it was 3.21 ± 0.21. In comparison to the conventional IV analgesics group, the VAS and DVAS were lower in the bupivacaine group, and this observation was statistically significant as the p-value calculated was <0.05.

**Table 1:** Postoperative pain assessment using a visual analogue scale. VAS: Visual analogue scale, IV: intravenous, SD: standard deviation

	Group				
	Bupivacaine		Conventional IV Analgesic		
	Mean	SD	Mean	SD	p
VAS 4hr	2.3	1.4	3.7	1.4	<0.001
VAS 8hr	2.0	1.3	3.0	1.1	<0.001
VAS 12hr	3.1	1.5	4.2	1.4	0.002
VAS 16hr	1.5	1.3	2.4	1.1	0.001
VAS 20hr	2.5	1.6	3.8	1.7	0.001
VAS 24hr	3.2	1.4	2.9	1.2	0.3
VAS 28hr	1.0	1.0	3.2	1.5	<0.001
VAS 32hr	3.5	1.6	3.1	1.1	0.2
VAS 36hr	2.9	2.0	3.8	1.3	0.02
VAS 40hr	1.8	1.3	2.4	1.4	0.04
VAS 44hr	2.8	1.5	3.6	2.0	0.047
VAS 48hr	3.1	2.0	2.7	1.4	0.3
VAS 52hr	1.0	0.8	3.0	1.4	<0.001
VAS 56hr	2.3	1.1	3.3	0.9	<0.001
VAS 60hr	2.9	1.1	3.1	1.1	0.3
	2.39	0.21	3.21	0.21	

**Table 2:** Postoperative pain assessment using a dynamic visual analogue scale. IV: Intravenous, DVAS: dynamic visual analogue scale, SD: standard deviation

	Group				
	Bupivacaine		Conventional IV Analgesic		
	Mean	SD	Mean	SD	p-value
DVAS 4hr	3.5	1.4	4.8	1.3	<0.001
DVAS 8hr	3.0	1.3	4.0	1.1	0.001
DVAS 12hr	4.3	1.4	5.2	1.4	0.004
DVAS 16hr	2.6	1.4	3.4	1.1	0.008
DVAS 20hr	3.6	1.6	4.8	1.7	0.001
DVAS 24hr	4.3	1.4	4.3	1.0	1
DVAS 28hr	2.2	1.1	4.4	1.4	<0.001
DVAS 32hr	4.5	1.6	4.1	0.9	0.3
DVAS 36hr	3.9	2.1	4.9	1.1	0.008
DVAS 40hr	2.8	1.3	3.5	1.4	0.02
DVAS 44hr	3.9	1.5	4.7	2.0	0.04
DVAS 48hr	4.1	2.0	4.0	1.2	0.7
DVAS 52hr	2.0	0.8	4.2	1.2	<0.001
DVAS 56hr	3.3	1.1	4.5	0.7	<0.001
DVAS 60hr	3.9	1.1	4.2	1.1	0.3
	3.46	0.21	4.33	0.14	

The study showed that in the first 24 hours, in the bupivacaine group, 14 patients had no rescue analgesic demands, whereas the remaining 26 patients had one demand each. In the conventional IV analgesics group, four patients had no demands, 17 patients had one demand each, and 19 patients had two demands each. As shown in Table 3, the bupivacaine group's rescue analgesic requirements were lower in the first 24 hours than those of the usual IV analgesics group (p<0.0001). For the next 24 hours, the local anaesthetic bupivacaine was effective; however, it was less compared to the first 24 hours which meant that in the first 48 hours postoperatively the bupivacaine group patients were in less pain compared to the conventional IV analgesics group. This allowed us to follow the Enhanced Recovery After Surgery (ERAS) protocol and mobilise the patient faster and decrease the occurrence of lung complications.

**Table 3:** Rescue analgesic demands. IV: Intravenous

		Group				
		Bupivacaine		Conventional IV Analgesic		
		Count	Column N %	Count	Column N %	p-value
Rescue analgesic (0-24 hrs)	0	14	35.0%	4	10.0%	<0.0001
	1	26	65.0%	17	42.5%	
	2	0	0.0%	19	47.5%	
Rescue analgesic (>24 hrs)	0	0	0.0%	8	20.0%	0.01
	1	19	47.5%	13	32.5%	
	2	21	52.5%	19	47.5%	

As shown in Table 4, an independent t-test revealed that the mean cumulative rescue analgesic use was higher in the traditional IV analgesics group compared to the bupivacaine group.

**Table 4:** Cumulative rescue analgesic requirement. IV: Intravenous, SD: standard deviation

	Group			
	Bupivacaine		Conventional IV Analgesic	
	Mean	SD	Mean	SD
Cumulative rescue analgesic requirement (Tramadol in mg)	125	35.35	200	0

The longer duration of surgery increases surgical stress. In this study, the duration of surgery was comparable in both groups eliminating any difference in outcome due to an increase in surgical stress. Surgical outcomes, such as time taken to start the patient orally and time taken to pass flatus, for a first bowel movement, and for independent ambulation, were noted to be statistically insignificant as demonstrated in Table 5. Hence, the study inferred that bupivacaine acted only as an analgesic locally reducing the pain without any additional impact on gut motility. It also concluded that the hospital stay was reduced due to better postoperative analgesia, in turn leading to lesser lung complications. Additionally, it was expected that placing a catheter in the subcutaneous plane would lead to more wound-related complications.

**Table 5:** Early surgical outcomes. IV: Intravenous, SD: standard deviation

	Group				
	Bupivacaine		Conventional IV Analgesic		
	Mean	SD	Mean	SD	p-value
Start orally (days)	3.6	1.0	3.8	1.1	0.4
Time to flatus (days)	3.9	.9	4.0	1.0	0.5
Time to first bowel movement (days)	5.9	1.7	6.4	1.5	0.2
Time to ambulate (days)	3.8	1.1	4.1	1.2	0.2

**Figure 2:**
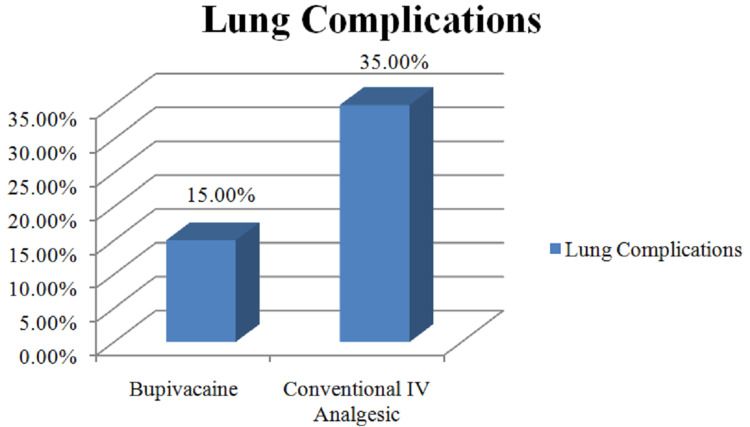
Postoperative lung complications. IV: Intravenous

In the present study, 35% of the patients getting conventional IV analgesics had postoperative lung complications, whereas only 15% of the patients from the bupivacaine group had postoperative lung complications as described in Figure 2. The postoperative patients do not optimally ventilate due to pain. The study hypothesised that better postoperative analgesia would avoid hypoventilation and in turn, reduce the occurrence of commonly seen lung complications after laparotomies. The postoperative lung complications include basal lung atelectasis, pleural effusion, and postoperative hypostatic pneumonia, which were recorded during data collection by the primary investigator, on the basis of the clinical condition of the patient, vital parameters, chest x-rays, and ultrasonography of the thorax. Hence, the presumption that adequate postoperative pain relief avoids hypoventilation thereby reducing the occurrence of lung complications that was supported by the findings of the present study.

**Figure 3:**
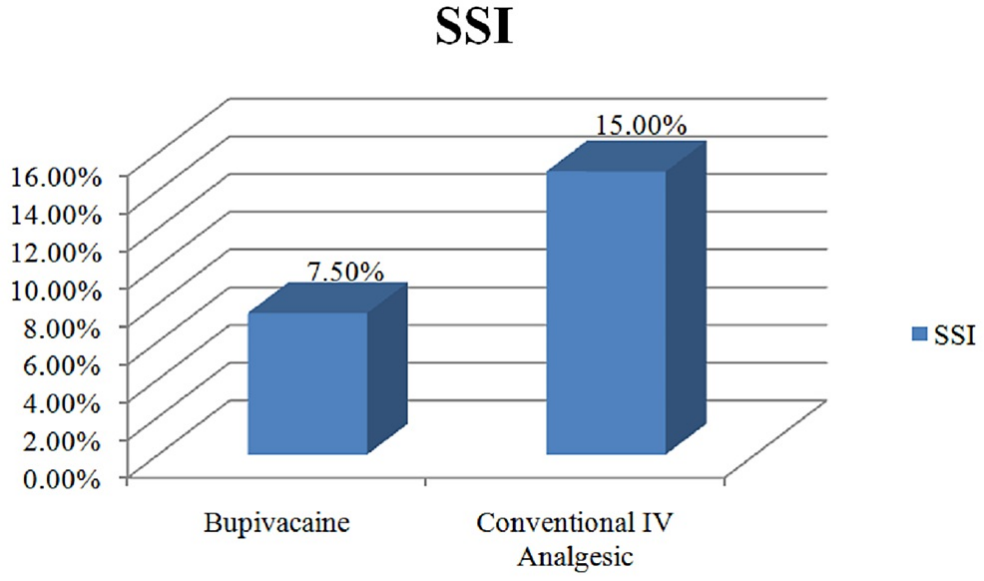
Distribution of surgical site infections. IV: Intravenous, SSI: surgical site infections.

In the present study, 15% of the patient getting conventional IV analgesics developed surgical site infections (SSI), whereas only 7.5% of the patient from the bupivacaine group had developed SSI as shown in Figure 3. 

In the bupivacaine group, the mean duration of hospital stays was noted to be 10.3 days compared to that of the conventional IV analgesics group which was 14.9 days. Regarding the number of days spent in the hospital, there was a statistically significant difference between the two groups (by the independent t-test, p<0.0001).

## Discussion

Multimodal analgesia is the use of more than one pharmacological class of analgesic drugs that target several pain pathway receptors with the aim of increasing analgesia and minimising class-specific side effects [9]. The first incidence of local anaesthetic solution irrigation at the surgical site was documented in a study done by Ewald Fulde and Walter Capelle in the early 1900s. The first wound infusion system was created in 1930 by two German surgeons who concentrated on establishing innovative techniques that allowed for both long-term pain control and early postoperative mobilization [10]. Local infiltration and continuous/intermittent infusion of anaesthetics into surgical wounds are now crucial aspects of postoperative pain management due to recent developments in multimodal analgesia [11].

Compared to other analgesic treatments like peripheral nerve blocks or epidural analgesia, wound instillation or infusion with local anaesthetic is an efficient, safe, and easy-to-perform method. At the end of the procedure, a catheter that was implanted by the surgeon is used to inject a local anaesthetic into the wound [7,12]. Both groups were comparable with respect to the mean age, gender, and co-morbidities distribution. The mean age was 48.02±15.7 (years) in the bupivacaine group with 77.5% being males and 22.5% being females. In the conventional IV analgesics group, 80% were males and 20% were females with the mean age being 52.35±18.1 (years). A study conducted by Khorgami et al. which included 60 patients, showed similar age distribution where the mean age was 43 years in the subcutaneous group, whereas it was 41 years in the interfascial group. However, paradoxical distribution was noted with the majority being females and the female-to-male ratio was 16:14 in the superfascial group versus 17:13 in the interfascial group [13].

The longer duration of surgery increases surgical stress. In this study, the average duration of surgery (in minutes) was noted to be 188.1 ± 55.2 and 175.3 ± 51.0 in the bupivacaine and conventional IV analgesics groups, respectively. Hence the duration of surgery was comparable in both groups eliminating any difference in outcome due to an increase in surgical stress. This study showed that in comparison to the conventional IV analgesics group, the mean VAS and DVAS were lower in the bupivacaine group. This led to conclude that, as compared to the commonly used IV analgesics, wound instillation with local anaesthetic bupivacaine offers superior postoperative analgesia. Similar results were noted in a prospective study conducted by Jonnavithula et al., which assessed the function of bupivacaine wound instillation through surgical drains for postoperative analgesia after modified radical mastectomy. The average time to the first dosage of a rescue analgesic was 14.6 hours for the bupivacaine group and 10.3 hours for the saline group. In comparison to patients who got saline, those who had wound instillation with 0.25% bupivacaine had higher postoperative analgesia [14].

However, a study comparing local continuous bupivacaine perfusion for pain relief with parenteral morphine infusion after laparotomy performed by Cheong et al. found no statistically significant differences in postoperative pain scores between the two groups, with the exception of pain scores at rest on the first postoperative day (P = 0.03) [15]. A study conducted by Fredman et al. demonstrated that recurrent wound instillation using an electronic patient-controlled analgesia device and a double-catheter system of 0.25% bupivacaine solution did not reduce pain or opioid requirement after surgery. The authors opined that the local anaesthetic dose was insufficient as well as that the drug did not spread evenly or rather, distributed unpredictably [16].

Infusion of local anaesthetics at the site of the wound has been suggested in some studies to reduce postoperative pain levels, but few other studies have found no such difference. Moreover, studies suggest that administering a placebo or a local anaesthetic to abdominal wounds results in identical opiate analgesic requirements. There may be controversy in the literature because of the large range of anaesthetic delivery methods utilized in various research. The study showed that the first 24 hours number of rescue analgesic demands was less in the bupivacaine group in the first 24 hours in comparison to the conventional IV analgesics group (p <0.0001). However, this did not hold true for the subsequent 24 hours. The most probable reason for the above occurrence could be receptor insensitivity or receptor saturation, due to which, after 24 hours the local anaesthetic bupivacaine was less effective.

In a study by Jonnavithula et al., there was no considerable difference between Groups B and S, although Group C (got no instillation) had more rescue analgesic demands than either Group B (bupivacaine) or Group S (saline) [14]. The early surgical outcomes, time taken to start the patient orally, time taken to first pass flatus, the first bowel movement, and independent ambulation, were found to be statistically insignificant. Hence, we inferred that bupivacaine acted only as an analgesic locally reducing the pain without any additional impact on gut motility. Therefore, our secondary objective of assessing the effect of wound instillation with local anaesthetic bupivacaine for optimal postoperative pain relief and thereby improving early surgical outcomes was not successful. However, a systematic review conducted by Liang et al., involving a total of 564 adults undergoing laparotomy for colorectal resection which is elective midline and comparing regular saline placebo to a continuous wound infusion of a local anaesthetic, demonstrated a reduced time to the first bowel movement [17].

There was a statistically significant difference in the number of patients developing postoperative lung complications, mainly by patients getting conventional IV analgesics (p=0.04). Hence, our presumption that adequate postoperative pain relief avoids hypoventilation thereby reducing the occurrence of lung complications such as atelectasis was supported by our findings. It was expected that placing a catheter in the subcutaneous plane would lead to more wound-related complications. However, in our study, 15% of the patient getting conventional IV analgesics developed SSI, whereas only 7.5% of the patient from the bupivacaine group had developed SSI with statistically significant results (p=0.03).

In comparison to the conventional IV, analgesics group the mean duration of hospital stays was considerably reduced in the bupivacaine group. This outcome was found to be statistically significant (by the independent t-test, p<0.0001). A comprehensive review conducted by Liang that revealed a shorter hospital stay in the local anaesthetic group compared to the control group backed these findings [17]. However, a trial by Khorgami et al. found that the mean hospital stay following surgery was equivalent in the two groups, with the suprafascial group staying in the hospital for 4.7 days and the interfascial group for 5.4 days [13].

The technique of bupivacaine instillation via a wound catheter is simple and efficient with no systemic toxicity, no anaesthetic or analgesic dosing errors, and almost negligible wound catheter-related complications. Only 3 out of 40 patients developed SSI which was managed with regular dressings. Also, it demonstrated the effectiveness of continuous wound catheters by enhancing analgesia, lowering the need and adverse effects of opioids, improving patient satisfaction, and shortening hospital stays.

The limitations of the present study involve a sample size not representative of the entire population, to completely eliminate the bias and the errors, a randomized controlled trial is recommended, pain level assessment was done only during the duration of stay in the hospital, long-term follow up of pain level was not evaluated, and cost analysis comparing the different modalities was not done.

## Conclusions

It is affordable, safe, and effective to administer bupivacaine via a catheter for postoperative analgesia to patients who have undergone midline laparotomies. This approach, when implemented correctly and with appropriate dosages, affords excellent outcomes with minimal adverse effects. Implementation of this technique into routine practice can significantly reduce the usage of systemic analgesics, in turn avoiding several of their adverse effects such as postoperative nausea and vomiting, constipation, respiratory depression, headache, dizziness, drowsiness, bradycardia, hypotension, and dependence. This approach can be applied as a sole means of providing postoperative analgesia in cases where the use of systemic analgesics or epidural anaesthesia is contraindicated, such as when NSAIDs are not suitable due to acute kidney injury or epidural catheter cannot be inserted in view of thrombocytopenia or hypotension.

In conclusion, bupivacaine wound instillation for midline laparotomy procedures was well tolerated and had no significant side effects. It provided far superior postoperative analgesia in comparison with systemic analgesics. If introduced in routine practice, it can potentially avoid systemic side effects of IV analgesics and significantly decrease the duration of hospital stays. As a result, this method of postoperative analgesia delivery can be added to the armory of multimodal analgesia.
